# Forkhead Box L1 Is Frequently Downregulated in Gallbladder Cancer and Inhibits Cell Growth through Apoptosis Induction by Mitochondrial Dysfunction

**DOI:** 10.1371/journal.pone.0102084

**Published:** 2014-07-10

**Authors:** Yiyu Qin, Wei Gong, Mingdi Zhang, Jiandong Wang, Zhaohui Tang, Zhiwei Quan

**Affiliations:** Department of General Surgery, Xinhua Hospital, Affiliated to School of Medicine, Shanghai Jiaotong University, Shanghai, China; Pontificia Universidad Catolica de Chile, Faculty of Medicine, Chile

## Abstract

**Background:**

Forkhead box L1 (FOXL1), considered as a novel candidate tumor suppressor, suppresses proliferation and invasion in certain cancers. However, the regulation and function of FOXL1 in gallbladder cancer (GBC) remains unclear.

**Methods:**

FOXL1 expression at mRNA and protein levels in GBC tissues and cell lines were examined by RT-PCR, immunohistochemistry and western blot assay. FOXL1 expression in GBC cell lines was up-regulated by transfection with pcDNA-FOXL1. The effects of FOXL1 overexpression on cell proliferation, apoptosis, migration and invasion were evaluated in vitro or in vivo. In addition, the status of mediators involved in migration, invasion and apoptosis was examined using western blot after transfection with pcDNA-FOXL1.

**Results:**

FOXL1 was frequently downregulated in GBC tissues and cell lines. Its higher expression is associated with better prognosis, while its lower expression is correlated with advanced TNM stage and poor differentiation. FOXL1 overexpression in NOZ cells significantly suppresses cell proliferation, migration and invasion in vitro and tumorigenicity in nude mice. FOXL1 overexpression disrupted mitochondrial transmembrane potential and triggered mitochondria-mediated apoptosis in NOZ cells. In addition, FOXL1 overexpression suppressed ZEB1 expression and induced E-cadherin expression in NOZ cells.

**Conclusion:**

Our findings suggested that dysregulated FOXL1 is involved in tumorigenesis and progression of GBC and may serve as a predictor of clinical outcome or even a therapeutic target for patients with GBC.

## Introduction

Gallbladder cancer (GBC) which represents the most common and aggressive type among biliary tract malignancies is characterized by non-specific presentation, late diagnosis and lack of effective treatment. Although recent advances have been made in the diagnosis and treatment, the outcomes of current therapies, such as surgery, chemotherapy and radiotherapy (alone or combined) have been proven to be dismal. It is associated with a poor prognosis with median survival duration of 4 months [1] and 5-year survival rate less than 10% [2]. Therefore, there is an urgent need to develop novel and effective therapy regimens for GBC patients. However, the limited knowledge on tumorigenesis of gallbladder cancer hinders the development of diagnosis and treatment for GBC. A better understanding of the molecular biology and carcinogenic mechanisms underlying development and progression of GBC may help to establish more effective treatments.

Forkhead box (FOX) proteins are a superfamily of transcription factors which share a highly conserved DNA-binding domain (the forkhead box or winged helix domain) control a variety of biological processes, including metabolism, differentiation, proliferation and apoptosis [3]. Since FOX proteins regulate these vital processes in growth and development, a gain or loss of FOX function unsurprisingly causes human genetic diseases including cancers. The dysregulation of several FOX subfamilies such as FOXO, FOXM, FOXP, FOXC and FOXA is implicated in tumorigenesis and progression of certain cancers [4]. Though FOX proteins share the highly conserved DNA-binding domain, their regulation and function vary significantly between families. FOX proteins have diverse functions and act as tumor suppressors or oncogenes during tumorigenesis and progression of various cancers. For instance, FOXO1 acts as a tumor suppressor which could induce apoptosis and cell cycle arrest in cancer cells [5]. In contrast to FOXO1, FOXM1 shows oncogenic activities and targeting FOXM1 induces apoptosis in cancer cells [6]. A growing body of evidence has convincingly shown that FOX proteins may represent attractive targets for therapeutic intervention against many cancers.

FOXL1 protein is a member of FOX superfamily which was initially discovered in the mesenchyme of the gastrointestinal tract. So far, the relationship between FOXL1 and gastrointestinal cancer including stomach, colon and pancreas has been investigated in several studies [7]–[8]. However, the biologic function of FOXL1 in GBC remains to be elucidated. In the present study, we investigated the significance of FOXL1 expression in patients with GBC in relation to clinical features and prognosis. We also conducted functional studies to evaluate the effects of FOXL1 overexpression on the proliferation, apoptosis, migration and invasion of GBC cells in vitro or in vivo. In addition, we preliminary investigated the expression of E-cadherin and Zinc-finger E-box binding homeobox 1 (ZEB1), which may contribute to the inhibitory effects of FOXL1 overexpression in GBC.

## Materials and Methods

### Ethics statement

The experiments involving human and animals were approved by the Ethical Committee of Xinhua hospital, Shanghai Jiaotong University School of Medicine. Written informed consents were obtained from all patients.

### Patients and tissue collection

35 of patients with GBC (13 men and 22 women, age range 51–71 yrs, mean age 61.9±5.4 yrs) treated with radical cholecystectomy (without prior radiotherapy or chemotherapy) between 2008 and 2013 at Department of general surgery, Xinhua hospital, Shanghai Jiaotong University School of Medicine were enrolled in this study. Tumor samples (n = 35) and adjacent nontumor samples (n = 12) were collected immediately after surgery and immersed in liquid nitrogen. According to the TNM staging system (AJCC, 7^th^, 2010), the tumors were staged (4 stage I, 12 stage II, 14 stage III, and 5 stage IV). The differentiation grade according to WHO criteria was as follows: 13 cases were well differentiated (WD), 15 moderately differentiated (MD) and 7 poorly differentiated (PD). The histological diagnosis of all GBC was confirmed by two pathologists.

### Immunohistochemistry

Frozen tissue samples were embedded in optimal cutting temperature (OCT) compound. 5-µm thick tissue sections of tumors and nontumor tissues were cut from tissue blocks, and then fixed in cold acetone at 4°C for 20 minutes. Tissue sections were incubated with 3% (v/v) H_2_O_2_ in methanol to eliminate endogenous peroxidase activity and then blocked with normal goat serum. Afterward, sections were processed for immunohistochemistry using antibodies against FOXL1 (1:100 dilution; Santa Cruz Biotechnology, Santa Cruz, CA) and secondary antibody (1:500 dilution; Santa Cruz Bio). Finally, sections were incubated with peroxidase complex and visualized by 3,3′-diaminobenzidine tetrachloride (DAB). All the immunostained sections were blindly reviewed by two independent pathologists. The intensity was evaluated using a 3-scale system (0, negative; 1, weak; 2, moderate; 3, strong). The percent positivity was also evaluated using a 3-scale system (0, <5%; 1, 5%–25%; 2, 25%–50%; 3, >50%). The overall quantitation for immunohistochemistry score was calculated by multiplying the score of staining intensity and score of percentage of positive cells. FOXL1 expression levels were ranked as follows: - (score 0–1), + (score 2–3), ++ (score 4–6) and +++ (score>6). According to the scores of FOXL1 immunostaining, GBC patients were classified into two groups: low expression (− or +) and high expression (++ or +++).

### Cell lines and culture conditions

Four human GBC cell lines GBC-SD, SGC-996, NOZ and OCUG-1 were used in this study. GBC-SD cell line was purchased from Cell Bank of the Chinese Academy of Sciences, Shanghai, China; SGC-996 cell line [9] was provided by Tumor Cell Biology Research Institute of Tongji University, Shanghai, China; NOZ and OCUG-1 cell lines were purchased from the Health Science Research Resources Bank, Osaka, Japan. Cells were cultured either in DMEM or RPMI1640 (Invitrogen, Carlsbad, CA, USA) supplemented with 1% penicillin/streptomycin (Invitrogen) and 10% (v/v) fetal bovine serum (HyClone, Logan, UT, USA) at 37°C in a humidified 5% CO_2_ incubator.

### Transfections

To upregulate the expression of FOXL1, pcDNA-FOXL1 (encodes FOXL1 cDNA) were constructed and transfected into GBC cells. The empty vector pcDNA3.1 (Invitrogen) was used as control. The cloning strategy and restriction maps of recombinant plasmids were shown in Figure S1. The transfection reagent Lipofectamine 2000 was used during transfection according to manufacturer’s instructions (Invitrogen). To obtain stable transfectants, the medium was replaced by fresh complete medium supplemented with 300 µg/ml geneticin (G418) after 48 h of transfection. The selective medium was refreshed every 3–4 days until G418-resistant clones can be identified. FOXL1 expression levels in cell lines were examined by RT-PCR and western blot assay.

### RNA isolation and RT-PCR analysis

Total RNA was extracted from frozen tissue samples and GBC cell lines using Trizol reagents kit (Gibco BRL, Gaithersburg, MD, USA). RNAs were converted into cDNA using a Takara RNA PCR kit (Takara Bio Inc, Dalian, China). The obtained cDNA was amplified using following procedure: 94°C (5 min), then 30 cycles at 94°C (30 s), 59°C (45 s), 72°C (45 s). The sequences of the primers for FOXL1 were as follows: Sense:cacggtactccacttccagt; Anti-sense:aacacttccctgaacccaca.

### Cell viability and colony formation assays

Water soluble tetrazolium (WST)-1 assay was performed to measure cell viability after transfection. 5×10^3^ of transfected cells per well were seeded into 96-well plates and cultured for 24, 48, 72, or 96 h. Afterward, cells were then incubated with WST-1 reagent for 2 h at 37°C. The absorbance at 450 nm was measured with the use of an automated microplate reader (Bio-Rad 5 Model 550, Bio-Rad, Hercules, CA, USA) and the growth curve was calculated.

For colony formation assay, 1×10^3^ of transfected cells per well were seeded in 6-well plates and grown in complete media at 37°C. After 14 days for culture, colonies were stained with crystal violet and the number of colonies was counted by automated colony counter (Alpha Innotech, San Leandro, CA, USA).

### Xenograft tumor experiments

Subcutaneous xenografts in nude mice were conducted to evaluate the effects of FOXL1 overexpression on cell growth and tumorigenicity in vivo. Male and Female Athymic (nu/nu) nude mice 6–8 weeks of age were obtained from Shanghai Laboratory Animal Center of Chinese Academy Sciences and housed under specific pathogen-free (SPF) condition. NOZ cells were stably transfected with pcDNA-FOXL1 or pcDNA3.1. Approximately 1×10^7^ of FOXL1-overexpressing cells or control cells were suspended in a total volume of 200 µL of 1/1 (v/v) PBS/Matrigel (BD Biosciences, San Diego, CA, USA) and then subcutaneously injected into flanks of the mice. About 4 weeks after the injection of tumor cells, visible subcutaneous tumor volumes were measured weekly with calipers and the tumor volumes in each group were calculated using the formula: volume = length×width^2^/2. All mice were sacrificed after 6 weeks of observation. Xenograft tumors were fixed in formalin and then embedded in paraffin. Tumor weight was measured and recorded.

### Flow cytometric analysis of apoptosis

The cell apoptosis in vitro was determined by flow cytometry using Annexin V-FITC Apoptosis Detection Kit (BD Bioscience) according to the manufacturer’s protocol. Briefly, 24, 48 and 72 h after transfection with pcDNA-FOXL1 or pcDNA3.1, cells were harvested, washed with PBS, and resuspended in 1×Binding Buffer. Afterward, cells were stained with FITC-Annexin V and PI for 15 min and analyzed by flow cytometry.

### TUNEL assay

Apoptosis in tissue sections from xenograft tumors was assessed by Terminal deoxynucleotidyl transferase dUTP nick end labeling (TUNEL) staining using In situ cell death detection kit, Fluorescein (Roche Diagnostics, Mannheim, Germany) following the manufacturer’s protocol. Briefly, xenograft tumor samples were deparaffinized using xylene and ethanol. Tissue sections were incubated with proteinase K solution for 15 min, followed by incubation with TUNEL reaction mixture. Afterward tissue sections were incubated with converter-POD and counterstained with 4′,6-diamidino-2-phenylindole (DAPI). The fluorescence was viewed by fluorescence microscopy using appropriate excitation and emission filters.

### Flow cytometric analysis of mitochondrial membrane potential

The alteration in mitochondrial transmembrane potential was determined by flow cytomery using the mitochondrial membrane potential sensitive fluorochrome dye JC-1. Briefly, NOZ cells were harvested 24, 48 and 72 h after transfection with pcDNA-FOXL1 or pcDNA3.1 and washed with PBS. Cells were incubated with JC-1 (10 µg/ml in PBS) at 37°C for 10 min. Stained cells were washed with PBS twice and analyzed by flow cytometry.

### Mitochondrial and cytosolic extract preparation

The amounts of mitochondrial cytochrome c and cytosolic cytochrome c were determined using mitochondrial extract and cytosolic extract respectively. Mitochondrial and cytosolic fractions were extracted from cultured cells by Mitochondria/Cytosol Fractionation Kit (Abcam, Cambridge, UK) according to the manufacturer’s protocol. Briefly, after washed with ice-cold PBS, transfected cells were incubated with cytosol extraction buffer mix containing dithiothreitol (DTT) and protease inhibitors on ice for 10 min. Cells were homogenized gently using a ice-cold glass homogenizer. The cell suspension was centrifuged at 3000 rpm at 4°C for 10 min. The supernatants were centrifuged again at 13,000 rpm for 30 minutes to separate intact mitochondria (pellets) and cytosolic fraction (supernatants). The pellets were resuspend with 100 µl of mitochondrial extraction buffer mix containing DTT and protease inhibitors and vortexed for 10 seconds. The mixture was saved as mitochondrial fraction.

### Western blot assay

Total proteins were extracted from NOZ cells 48 h after transfection with pcDNA-FOXL1 or pcDNA3.1. The protein content was quantified by Bradford protein assay. 40 µg of protein was separated by 10–12% SDS-PAGE and electrotransferred to PVDF membranes (Millipore, Billerica, MA, USA). The membrane was blocked with 5% non-fat dry milk, and probed with corresponding primary antibodies (Santa Cruz Bio) overnight at 4°C, followed by incubation with horse radish peroxidase-coupled secondary antibodies (Santa Cruz Bio). Horseradish peroxidase activity was visualized using enhanced chemiluminescence (ECL) kit (Pierce, Rockford, IL, USA) and exposed to X-OMAT-Blue film (Kodak, Rochester, NY, USA).

### Cell migration and invasion assay

Cell migration and invasion assay were performed using a 24-well transwell chamber (Corning Inc, Corning, NY, USA) with 8 µm pores according to the manufacturer’s protocol. The transfected cells were seeded in the uncoated (migration assay) or matrigel-coated (invasion assay) upper chamber compartement with serum-free RPMI1640. RPMI1640 supplemented with 10% fetal bovine serum were placed in the lower chambers as chemoattractant. 24 h after incubation, cells on the upper side of the filter membrane were removed with a cotton swab. Cells that stick to the lower surface of the membrane were fixed with 4% paraformaldehyde for 30 minutes and then stained with crystal violet for 20 minutes.

### Statistical analysis

Data were presented as mean ± SD. Statistical analysis was conducted with SPSS 11.0 (SPSS Inc, Chicago, IL, USA). The differences between groups were evaluated using Student’s t test or One-Way ANOVA or Kaplan–Meier log-rank or chi-sqaured test. The statistical significance was defined as P<0.05.

## Results

### FOXL1 expression in GBC and its relationship to clinicopathological variables

**Figure 1:**
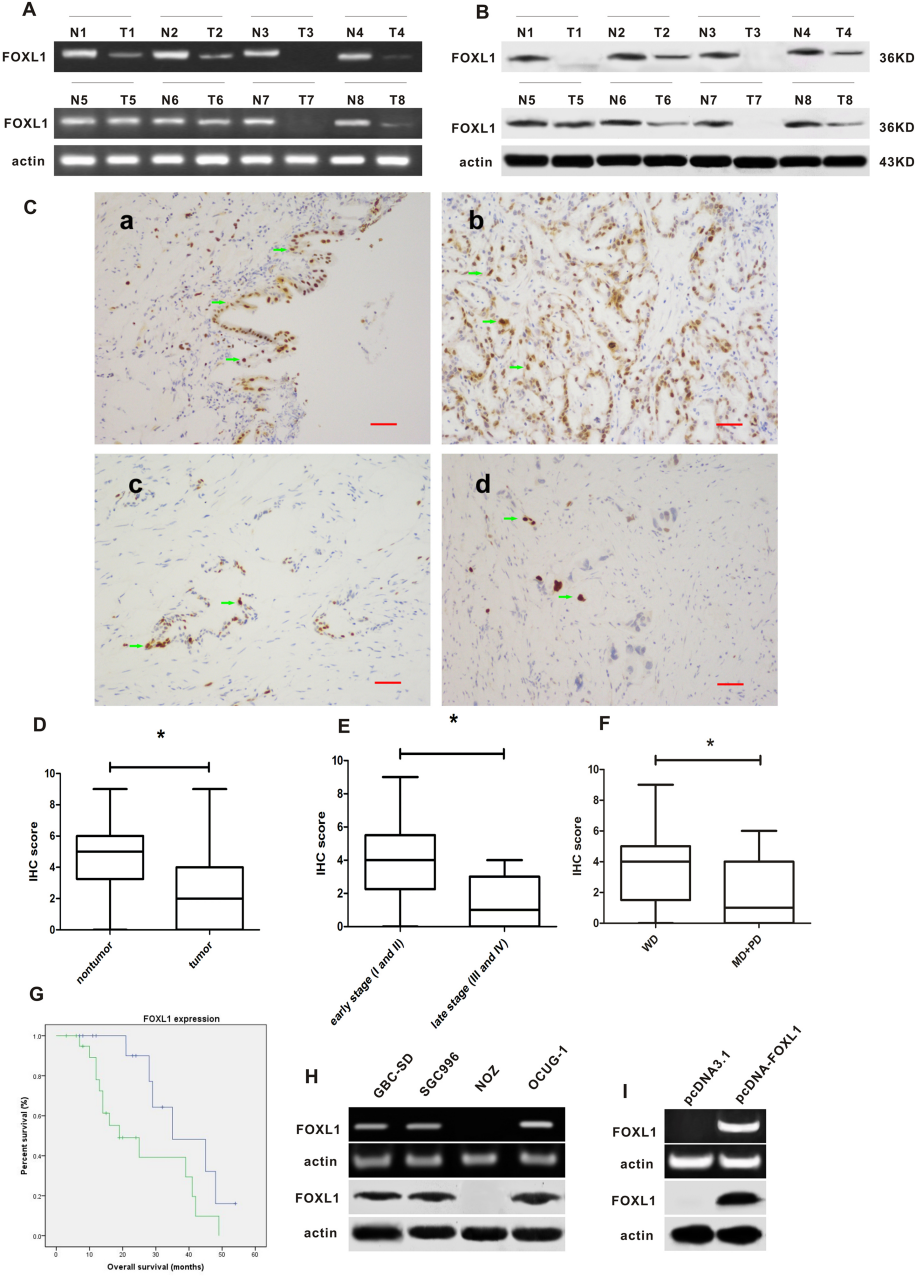
FOXL1 expression in GBC tissues and cell lines. (**A**) FOXL1 mRNA expression in GBC tissues and matched nontumor tissues. T: GBC tissues; N, matched nontumor tissues. (**B**) FOXL1 protein expression in GBC tissues and matched nontumor tissues. (**C**) Immunohistochemistry analysis of FOXL1 in GBC tissues and matched nontumor tissues. The magnification is 200× and the scale bar is 50 µm. **a**: Positive staining of FOXL1 in nontumor tissues; **b**: Positive staining of FOXL1 in well differentiated (WD) tumors; **c**: Positive staining of FOXL1 in moderately differentiated (MD) tumors; **d**: Positive staining of FOXL1 in poor differentiated (PD) tumors; (**D**) IHC score of GBC tissues and nontumor tissues (P<0.05). (**E**) IHC score of early TNM stage (I and II) tumors and late TNM stage (III and IV) tumors (P<0.05). (**F**) IHC score of WD tumors and MD+PD tumors (P<0.05). (**G**) Kaplan-Meier curves based on FOXL1 expression levels by immunohistochemistry in GBC. The patients with high expression of FOXL1 have better outcome as compared to those with low expression of FOXL1 (P<0.05). (**H**) The mRNA and protein expression of FOXL1 in GBC-SD, SGC996, NOZ and OCUG-1 cell lines. (**I**) Transfection with pcDNA-FOXL1 restored expression of FOXL1 in NOZ cells. *indicates significant difference.

FOXL1 mRNA and protein were examined in tumor tissues and nontumor tissues by RT-PCR, western blot and immunohistochemistry analysis, respectively. As shown in Figure 1A and B, FOXL1 mRNA and protein levels were decreased in cancer tissues as compared with matched nontumor tissues. Moreover, 2 of tumor samples lack FOXL1 mRNA expression and 3 of tumor samples lack protein expression.

Immunohistochemical analysis showed that FOXL1 protein was mainly localized in nuclear of cancer cells and normal epithelial cells (Figure 1C). FOXL1 protein was abundantly expressed in normal tissues, but was relatively less expressed in cancer tissues. The score of FOXL1-positive immunostaining in GBC tissues was much lower than that in normal tissues (P<0.05, Figure 1D). FOXL1 expression was significantly higher in tumors with earlier stage (I and II) as compared with those with late stage (III and IV) (P<0.05, Figure 1E). Difference in score of FOXL1-positive immunostaining was also measured between tumor grades (WD vs PD+MD) and was found to be significant (P<0.05, Figure 1F), with higher FOXL1 scores in WD groups. In addition, a higher level of FOXL1 gene expression was associated with better prognosis (P<0.05, Figure 1G).

The endogenous FOXL1 mRNA and protein were detectable in GBC-SD, SGC996 and OCUG-1 cell lines, but not in NOZ cell line which is therefore chosen for subsequent experiments (Figure 1H). Transfection with pcDNA-FOXL1 restored the mRNA and protein levels of FOXL1 in NOZ cell line (Figure 1I).

### FOXL1 overexpression suppresses proliferation of NOZ cell line in vitro and in vivo

**Figure 2:**
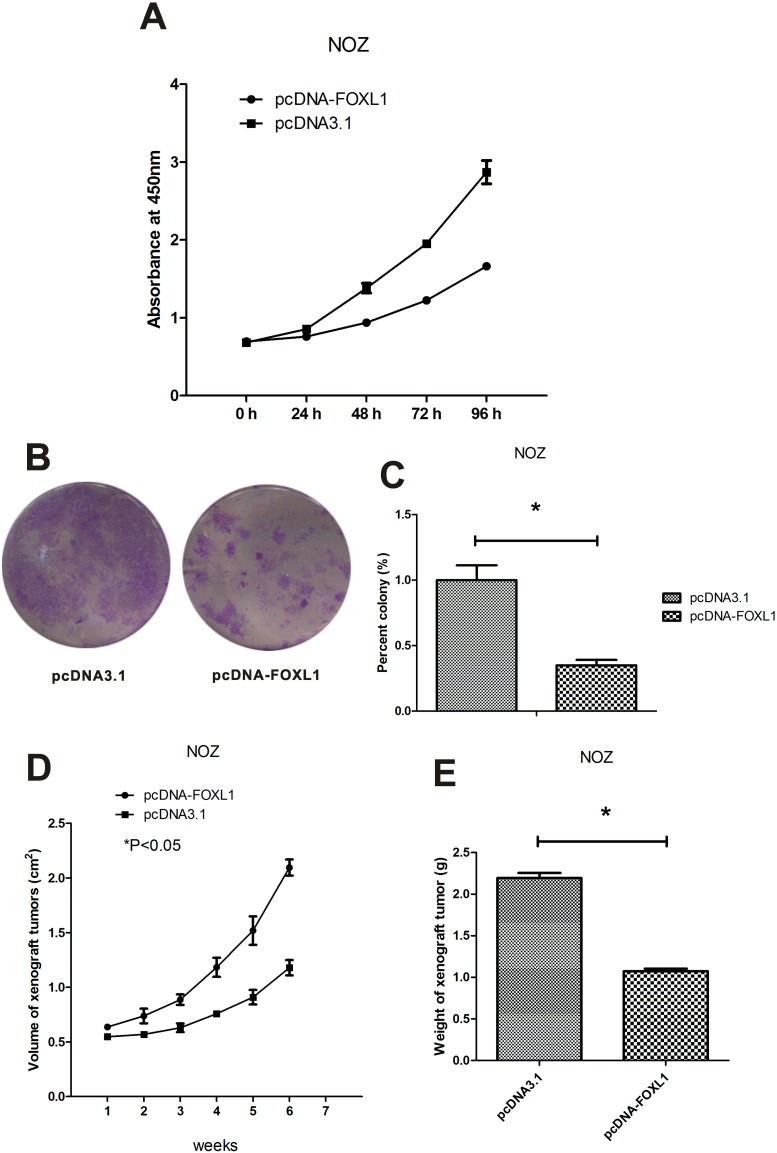
FOXL1 overexpression inhibited growth of NOZ cells in vitro and in vivo. (**A**) The growth of NOZ cells was significantly inhibited after transfection with pcDNA-FOXL1 (P<0.05). (**B**) Colony formation by NOZ cells. The results shown are representative of three experiments. (**C**) Transfection with pcDNA-FOXL1 significantly reduced the number of colony in NOZ cells (P<0.05). (**D** and **E**) FOXL1 overexpression reduced the volume and weight of xenograft tumor (P<0.05). *indicates significant difference.

The in vitro effect of FOXL1 overexpression on the proliferation and growth of NOZ cells was assessed by WST-1 assay. Overexpression of FOXL1 significantly inhibited cell proliferation in NOZ cells (P<0.05; Figure 2A). And this growth inhibition was time-dependent.

The more long-term inhibitory effects of FOXL1 overexpression on cell proliferation was assessed by colony formation assay. A significant decrease in colony number was observed in FOXL1-overexpressing cells compared with control cells (P<0.05, Figure 2B and C).

To further investigate the role of FOXL1 in tumorigenicity and growth of GBC in vivo, xenografts in nude mice were established by subcutaneous implantation of FOXL1 overexpressing and control cells. Xenografts formed by FOXL1 overexpressing cells grew at a much slower rate than those formed by control cells. FOXL1 overexpression in vivo induced a significant reduction in both tumor volume (P<0.05, Figure 2D) and tumor weight (P<0.05, Figure 2E). These findings suggested that FOXL1 suppresses the growth of GBC cells both in vitro and in vivo.

### FOXL1 overexpression promoted apoptosis in NOZ cell line in vitro and in vivo

**Figure 3:**
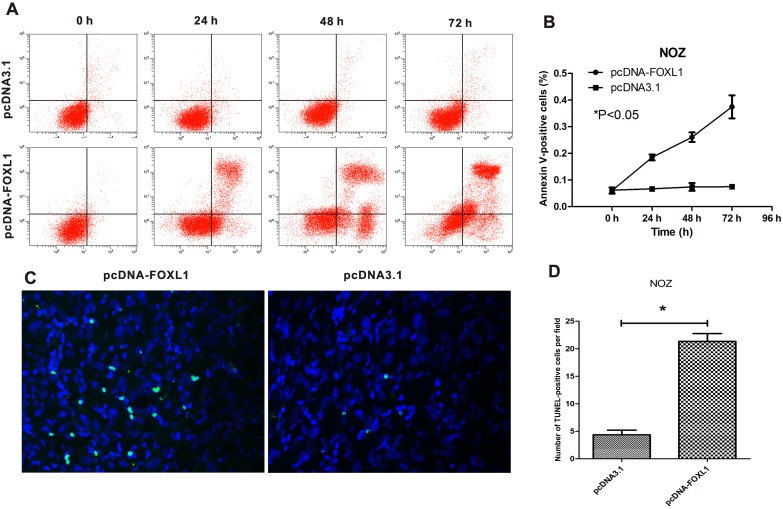
FOXL1 overexpression promoted apoptosis in vitro and in vivo. (**A**) Apoptosis of NOZ cells in vitro was examined by Annexin V/PI double staining. Cells that stain negative for both Annexin V and PI are viable cells (lower left). Cells that stain positive for Annexin V and negative for PI are undergoing early apoptosis (lower right). Cells that stain positive for both Annexin V and PI are either in the end stage of apoptosis, or undergoing necrosis (upper right). The results shown are representative of three experiments. (**B**) The number of apoptotic NOZ cells (including early apoptosis, late apoptosis and necrosis) was significantly increased after transfection with pcDNA-FOXL1 (P<0.05). (**C**) Apoptosis of NOZ cells in vivo was determined using TUNEL assay. Apoptotic cells are visualized as intensely green fluorescent cells. The number of apoptotic cells was recorded under high-power fields (400×). Localization in nuclei was visualized by counterstaining with DAPI (blue). (**D**) FOXL1 overexpression significantly promoted apoptosis of NOZ cells in vivo (P<0.05). *indicates significant difference.

Induction of apoptosis by FOXL1 overexpression in vitro and in vivo was examined using Annexin V/PI assay and TUNEL assay, respectively. As shown in Figure 3A and B, a significant increase in the proportion of apoptotic NOZ cells (early apoptosis plus late apoptosis) was observed after transfection with pcDNA-FOXL1 (P<0.05). And the proportion of late apoptotic or necrotic cells increased in a time-dependent manner (started from 24 h and reached the peak at 72 h).

TUNEL assay showed that FOXL1 overexpression promoted apoptosis of NOZ cells in xenograft tumors. There were fewer TUNEL-positive cells in xenograft tumors formed by control cells. By contrast, the mean number of TUNEL-positive cells significantly increased in xenograft tumors formed by FOXL1 overexpressing cells (P<0.05, Figure 3C and D).

### FOXL1 overexpression induced mitochondrial transmembrane depolarization and cytochromec-mediated apoptosis

**Figure 4:**
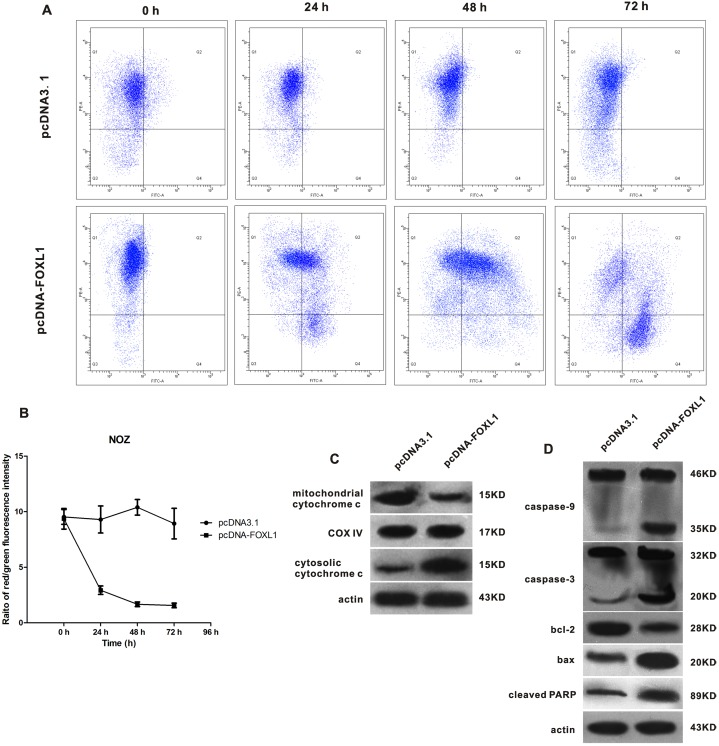
FOXL1 overexpression induced mitochondrial dysfunction and caspase-mediated apoptosis. (**A**) The loss of mitochondrial membrane potential (ΔΨm) is indicated by a decrease in the red/green fluorescence intensity ratio. X axis indicates green fluorescence intensity; Y axis indicates red fluorescence intensity. The results shown are representative of three experiments. (**B**) The proportion of red fluorescence-positive cells decreased while the proportion of green fluorescence-positive cells increased after transfection with pcDNA-FOXL1 (P<0.05). (**C**) Western blot analysis showed the level of cytosolic cytochrome c markedly increased, while the level of mitochondrial cytochrome c markedly decreased. (**D**) The levels of cleaved caspase-9, 3 and PARP and bax were elevated, while the level of bcl-2 was decreased. *indicates significant difference.

Loss of mitochondrial membrane potential (ΔΨm) indicates the initiation and activation of some apoptotic cascades. In the present study, ΔΨm was examined by JC-1 staining. Loss of ΔΨm was determined by the change in JC-1 fluorescence from red to green. As shown in Figure 4A and B, the proportion of red fluorescence-positive cells decreased while the proportion of green fluorescence-positive cells increased after transfection with pcDNA-FOXL1 (P<0.05), suggesting FOXL1 overexpression induced an apparent reduction in mitochondrial membrane potential.

Mitochondrial and cytosolic cytochrome c was examined by western blot assay. The level of cytosolic cytochrome c was markedly elevated, while the level of mitochondrial cytochrome c was markedly decreased 48 h after transfection with pcDNA-FOXL1, suggesting that FOXL1 overexpression promoted cytochrome c release from mitochondria to cytosol (Figure 4C). Cytochrome c oxidase subunit IV (Cox IV) and β-actin were used as control for mitochondrial protein and cytosolic protein, respectively. Furthermore, we found pcDNA-FOXL1 induced an increase in the Bax/Bcl-2 ratio, which may trigger the release of cytochrome c from mitochondria to cytosol.

The effects of FOXL1 overexpression on caspases activation in NOZ cells was further examined by Western blot. We found that cleavage of caspase-9 and -3 was significantly increased 48 h after transfection with pcDNA-FOXL1. In addition, the cleavage of poly (ADP)-ribose polymerase (PARP) which is the hallmark of apoptosis was also increased remarkably after transfection with pcDNA-FOXL1 (Figure 4D). These findings suggested that FOXL1 overexpression activated caspase cascades in NOZ cells.

### FOXL1 suppressed migration and invasion of NOZ cells and induced E-cadherin expression

**Figure 5:**
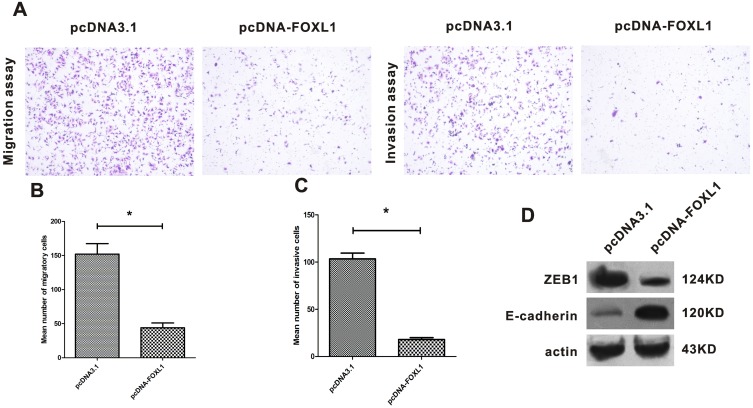
FOXL1 suppressed migration and invasion of NOZ cells and induced E-cadherin expression. (**A**) The number of migratory and invasive cells stained with crystal violet reflected their migratory and invasive capacity. The numbers of migratory or invasive NOZ cells per visual field were counted under microscopy after crystal violet staining (×100). (**B** and **C**) Transfection with pcDNA-FOXL1 significantly inhibited migration and invasion of NOZ cells (P<0.05). (**D**) ZEB1 expression was suppressed and E-cadherin expression was induced by FOXL1 overexpression. *indicates significant difference.

Given that the association of FOXL1 expression with clinical stage and lymph node metastasis of GBC had been demonstrated above, it is expectedly that FOXL1 may play a role in migration and invasion of GBC. Therefore, we next evaluated the role of FOXL1 in the migration and invasion of GBC. As shown in Figure 5A–C, transfection with pcDNA-FOXL1 induced a significant decrease in the numbers of migratory and invasive NOZ cells in comparison with the control groups (P<0.05), suggesting FOXL1 overexpression impaired the migratory and invasive abilities of NOZ cells. Furthermore, we found E-cadherin protein level was significantly elevated 48 h after transfection with pcDNA-FOXL1, while the level of ZEB1 protein was significantly reduced (Figure 5D).

## Discussion

The roles of Forkhead family members such as FOXOs, FOXMs and FOXPs in cancers have been widely investigated. However, there are few researches on the regulation and function of FOXL1 in cancers. In the present study, the role of FOXL1 in growth, apoptosis, migration and invasion of GBC cell was preliminarily studied. We found the levels of FOXL1 mRNA and protein were significantly decreased in GBC tissues as compared with matched nontumor tissues. Besides GBC, a decline in expression of FOXL1 was also observed by other researchers in pancreatic tumors [10], [11]. In addition, FOXL1 expression was negatively correlated with advanced TNM stage and poor differentiation of GBC. Moreover, a higher FOXL1 expression was associated with better prognosis in patients undergoing surgery for GBC. These findings suggested that downexpression of FOXL1 may be involved in tumorigenesis and progression of GBC and FOXL1 may serve as a useful predictor of prognosis for those patients undergoing surgery. Similar results were also observed in myeloma and pancreatic cancer [8], [12]. However, the mechanisms for downexpression of FOXL1 expression in these cancers have not been elucidated yet. Several mechanisms such as chromosomal deletion [13], chromosome translocations [14], [15], promoter methylation [16], alteration in upstream regulators [17], [18] and post-translational modifications [4] have been demonstrated to contribute to deregulation of Fox factors. Based on the present results, it is hard to say which mechanism was responsible for the downregulation of FOXL1 in GBC. Further investigation on genetic and epigenetic mechanisms for deregulated FOXL1 gene expression ought to be carried out to clarify the issue.

The correlation of higher FOXL1 expression with early stage and better prognosis suggested its potential suppressive role in GBC cell growth and progression. Hence we carried out a series of functional studies to evaluate the effect of FOXL1 on the growth, apoptosis, migration and invasion of GBC cell line.

Overexpression of FOXL1 significantly inhibited growth of NOZ cells in vitro and in vivo, and this growth inhibition may be attributed to the induction of apoptosis by FOXL1 overexpression. Apoptosis is a biological process involving a genetically programmed series of events leading to the death of a cell. During this process, several key events occur in mitochondria, including the release of cytochrome c from mitochondria to cytoplasm and the loss of mitochondrial transmembrane potential (ΔΨm) [19], [20]. ΔΨm is an important parameter of mitochondrial function and has been used as an indicator of viable cells. Bcl-2 family consisting of pro- apoptotic and anti-apoptotic members regulates the mitochondrial pathway of apoptosis by controlling the permeabilization of the outer mitochondrial membrane and the opening of voltage-dependent anion channel (VDAC). Pro-apoptotic protein such as Bax accelerates the opening of VDAC, whereas the anti-apoptotic protein Bcl-x(L) closes VDAC by binding to it directly. Increase in the Bax/Bcl-2 ratio may lead to disruption of ΔΨm and opening of VDAC. Subsequently, mitochondrial cytochrome c may translocate to the cytosol through VDAC and initiate the process of apoptosis [21]. In the present study, we found that ΔΨm was disrupted, the bax/bcl-2 ratio was elevated, and cytochrome c was released from mitochondria to cytosol after transfection with pcDNA-FOXL1. In addition, caspase-9 and -3, both of which function downstream of cytochrome c release [22] were both activated. Moreover, PARP, the substrate of caspase-3 was also activated. The cleavage of PARP was the hallmark of apoptosis [23]. These findings suggested that FOXL1 overexpression promoted mitochondrial apoptotic pathway.

E-cadherin which is recognized as a tumor suppressor gene in many types of cancer, is frequently downregulated in gallbladder cancer [24]. Absence or downregulation of E-cadherin is considered as a key initial step during EMT which facilitates migration and invasion of tumor epithelial cells into surrounding tissues [25]. ZEB1, one of EMT-inducing transcriptional repressors, binds the E-boxes in the E-cadherin promoter and represses E-cadherin transcription [26]. A recent study revealed that FOXL1 could bind to the promoter of ZEB1 and suppresses ZEB1 transcription [8]. So it is reasonable to suppose that overexpression of FOXL1 may induce the E-cadherin expression. To test the hypothesis, we examined the expression of ZEB1 and E-cadherin after transfection with pcDNA-FOXL1. As expected, overexpression of FOXL1 suppressed ZEB1 expression, which hence induced the expression of E-cadherin. These findings further provided an explanation for reduced migration and invasion of NOZ cells after transfection with pcDNA-FOXL1.

In addition, the role of E-cadherin in apoptosis has been investigated in several studies. E-cadherin overexpression could induce apoptosis, increases sensitivity to epidermal growth factor receptor inhibitors in lung cancer cell lines [27]. The induction of apoptosis by E-cadherin expression may be mediated by its suppressive activities on Bcl-2 expression [28]. Based on the fact that FOXL1 overexpresion decreased the expression of Bcl-2 and induced mitochondrial dysfunction, it is suggested that E-cadherin may play a role in induction of apoptosis by FOXL1 overexpression.

In summary, our findings suggested for the first time that FOXL1 may play a tumor suppressor role in GBC and downregulation of FOXL1 is a common event in GBC. Low FOXL1 expression is associated with advanced TNM stage, lymph node metastasis and poorer prognosis of GBC. Overexpression of FOXL1 inhibited growth, migration and invasion and induced apoptosis in GBC cells. Reactivation of E-cadherin may contribute to these inhibitory activities of FOXL1. FOXL1 may serve as a potential therapeutic target for patients with GBC. Admittedly, there remain several important issues to be clarified in further studies.

## Supporting Information

Figure S1 **Cloning strategy and restriction maps of plasmids pcDNA3.1 (A) and pcDNA-FOXL1 (B).**
(TIF)
