# Supplementary figures and images for: Forkhead Box L1 Is Frequently Downregulated in Gallbladder Cancer and Inhibits Cell Growth through Apoptosis Induction by Mitochondrial Dysfunction
Source: PLoS One. 2014 Jul 10;9(7):e102084. doi: 10.1371/journal.pone.0102084 (PMC4092092; doi:10.1371/journal.pone.0102084)

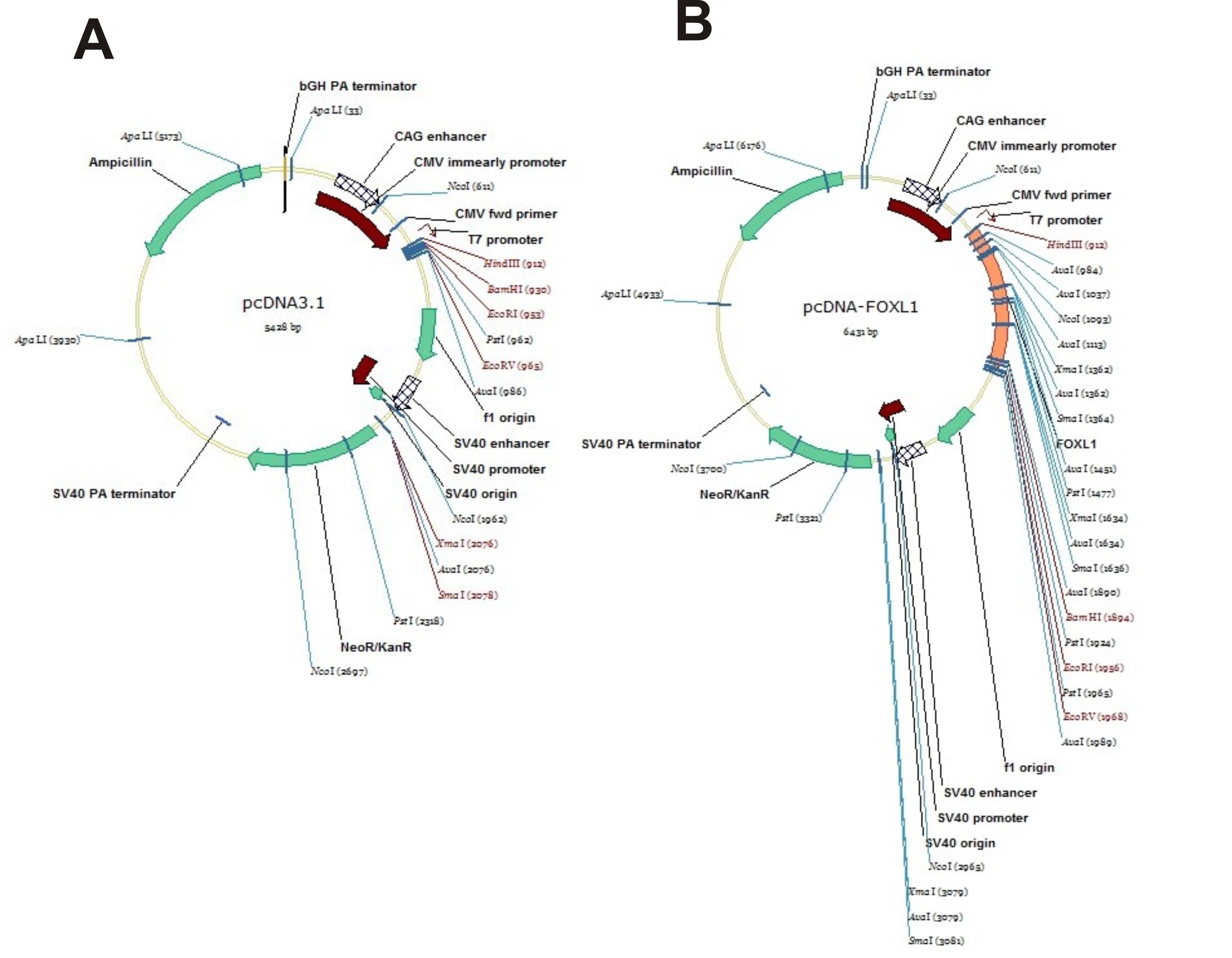

Supplement: Figure S1 — Cloning strategy and restriction maps of plasmids pcDNA3.1 (A) and pcDNA-FOXL1 (B). (TIF) [file pone.0102084.s001.tif]
